# Levels of 25 cytokines in the first seven days of life in newborn infants

**DOI:** 10.1186/1756-0500-6-547

**Published:** 2013-12-20

**Authors:** Setyadewi Lusyati, Christian V Hulzebos, Jantien Zandvoort, Pieter JJ Sauer

**Affiliations:** 1 Department of Pediatrics, Harapan Kita Women and Children Hospital, Jakarta, Indonesia; 2 Department of Pediatrics, Beatrix Children Hospital, University Medical Center Groningen, Groningen, The Netherlands; 3 Harapan Kita Women and Children Hospital, S. Parman Kav 87 Slipi, West Jakarta, Jakarta 14012, Indonesia

**Keywords:** Cytokines, Chemokines, Preterm-infants, Term-newborn

## Abstract

**Background:**

Novel methods for cytokine analysis allow for the simultaneous measurement of 25 cytokines in 50 μL serum or plasma. Data on values of most of these cytokines in non-infected newborn infants are lacking. We analyzed levels of 25 cytokines in the first week of life in non-infected preterm and term infants and related them to gestational age.

**Findings:**

During the first week after birth, no trend over time was found in any of the cytokines, except for IL-1Ra and IL-6 where higher values were found in the first four hours. Between 24 and 72 hrs levels of IL-1Ra, IL-2, IL-8, IL-12, IL-13, IL-15, IL-17, IFNγ, MIP-1a, MCP-1, TNFα were lower in infants born after 30-32 wks compared to infants ≥36 wks; levels of IL-6, IL-10, IP-10 were lower in preterm infants of both 30–32 and 33–36 weeks. No difference between groups for any of the levels was found for IL-1b, IL-2r, IL-4, IL-5, IL-7, IFNa, MIP-1b, GM-CSF, Eotaxin and RANTES.

**Conclusions:**

Levels of 25 interleukines are stable in the first week of life in non-infected infants. Infants born after 30-32 wks showed lower levels of fourteen cytokines compared to infants born after more then 36 wks. This indicates a lower stimulation or activation of Th-1 cells, monocytes and dendritic cells in these infants.

## Introduction

Infections are an important cause of morbidity and mortality in preterm as well as term infants [1]. Clinical signs that might indicate the presence of an infection are not specific. Antibiotics are used frequently when an infection is suspected. The widespread use of antibiotics has a number of important side-effects, including the occurrence of bacterial resistance and the development of an abnormal gastro-intestinal flora [2]. Reliable methods to detect an infection in newborn infants therefore are needed. Cytokines and chemokines have been evaluated for this purpose. However, they are still not used in clinical practice. Studies found higher levels of IL-1Ra, IL-6, IL-8, IP-10 and MIP-1a in infants with a proven infection, but there was overlap in results between infants with and without an infection [3-11]. Data on cytokine levels in the first week of life in healthy, non-infected infants are very limited [12-14]. For some of the cytokines and chemokines an increase after birth was found, while for others a decrease was observed [15,16]. Another unresolved question is the effect of gestational age on cytokine levels. In most studies only preterm infants are included.

Studies conducted so far included a limited number of interleukines. Using the Luminex array it has become possible to measure up to 25 cytokines and chemokines at the same time in only 50 μl of plasma or serum. A number of these 25 cytokines have not been evaluated as potential markers for neonatal infection. Before a cytokine can be used in daily practice it is essential to know levels in non infected infants and to know if these level are related to gestational age. The aim of this study is to measure sequentially in the first week of life 25 cytokines and chemokines in non-infected infants with different gestational ages admitted to our neonatal intensive care unit.

## Research methods

### Subjects

This study is part of a large prospective study on interleukin levels conducted at the NICU of Harapan-Kita Women and Children Hospital, Jakarta, Indonesia from October 2007 till October 2009. For the present study patients were selected by consecutive sampling when they were inborn and showed no clinical signs compatible with an infection during the first seven days of life other than mild respiratory problems for which N-CPAP was given with 21% of FiO_2_. As CPAP is used very frequent in our unit for mild respiratory problems in infants without other symptoms, the use of CPAP was allowed. The blood culture, taken soon after birth, had to be negative. As is practice in our NICU, all infants admitted with -mild- respiratory problem, were treated with broad spectrum antibiotics (Ampicillin-sulbactam and an aminoglycoside). The study was approved by the Research Ethical Committee, Harapan Kita Women and Children Hospital, Jakarta. Informed consent was obtained from the parents when infants were included.

### Routine laboratory and cytokine measurement

Blood was taken on admission for clinical purposes, including a blood culture. An additional 0.3 ml was taken for cytokine levels. Thereafter the same amount of blood was taken at 4, 12, 24 hrs and at day 2, 3, 4, 5, 6 and 7 together with blood taking for clinical purposes. In infants with a gestational age of 32 weeks and less the blood sampling for this study was stopped after day three, as it is was considered not ethical to take blood in these tiny infants for study purposes only. The blood sample was centrifuged immediately, 50 μl of serum was sampled and stored at -80°C. Samples were shipped on dry ice to the University Medical Center Groningen, The Netherlands, where they were analyzed. Sera were thawed and analysed using Invitrogen’s Multiplex Bead Immunoassay. In a 96 well plate samples were prepared by adding beads of defined spectral properties which were conjugated to protein-specific capture antibodies, incubation buffer to bind cytokines to the protein-specific capture antibodies and biotinylated detector antibodies. Finally streptavidin conjugated to the light-sensitive fluorescent protein R-Phycoerythrin was added and cytokine concentrations were analyzed with the Luminex detection system (Luminex Corp., Austin, Texas) using the programme StarStation 2.3. By monitoring the spectral properties of the beads and the amount of associated R-Phycoerythrin (RPE) fluorescence, the concentration of proteins was determined. The cytokines/chemokines of IL-1b, IL-1Ra, IL-2, IL-2r, IL-4, IL-5, IL-6, IL-7, IL-8 (CXCL-8), IL-10, IL-12, IL-13, IL-15, IL-17, TNFα, IFNa, IFNγ, IP-10 (CXCL-10), MIP-1a (CCL-3), MIP-1b (CCL-4), Eotaxin, Rantes (CCL-5), GM-CSF, MIG, MCP-1 (CCL-2) were measured.

### Statistical analysis

To study the effect of gestational age the infants were divided in three groups, 30-32 wks, 33-35 wks and ≥36 wks. Differences between groups were analyzed by Non Parametric statistic (Mann–Whitney U test). The trend over time was evaluated using the Rank- Spearman test. To correct for multiple comparisons, the significance for all analysis was set at p < 0.01.

## Findings

We studied thirty-four non-infected inborn infants admitted at our NICU of Harapan Kita Women and Children Hospital. Clinical characteristics of these infants are given in Table 1. None of the infants did have perinatal asphyxia, as indicated by an Apgar score of at least 5 after 5 minutes. The majority of infants were born by caesarean section. One mother showed fever before birth. As is practice in our hospital, the majority of mothers received antibiotics just before birth, mainly because of the caesarean section. The blood culture was negative in all infants, except for one preterm infant who showed a positive blood culture with Coagulase positive staphylococcus, this was considered a contamination.

**Table 1:** Characteristics between study groups

**Characteristics**	**30 - 32 weeks**	**33 – 35 weeks**	**≥ 36 weeks**
	**(n = 11)**	**(n = 14)**	**(n = 9)**
GA (wks). Median (25–75th)	32 (31 – 32)	34(33 – 34)	38 (36.5 – 38.5)
BW (gram), mean (range)	1557 (1415 – 1662)	1871 (1671 – 2091)	2586 (1800 – 3232)
C-Section	10	10	9
Gender: Male	3	7	6
AS less than 5 at 5 min	0	0	0
Clinical Amnionitis^@^	4	2	2
CPAP (duration (days))	10 (3)	11 (3)	8 (3)
Leucocyte < 5000 or > 30.000/mm^3^	1	0	2
Thrombocyte < 150.000 or > 600.000/mm^3^	1	3	2
Day antibiotics (median)	5 (3 – 12	4 (4 – 7)	4 (4 – 6)
Length of hospitalization at NICU (mean)(days)	16 (8 – 23)	7 (4 – 10)	8 (6 – 9)

^@^: PROM more than 12 hours; decceleration CTG; Maternal fever ≥ 38°C; Maternal leucocytes > 15.000/mm^3^.

Most of cytokines and chemokines were detected in almost all infants. The results of all interleukin levels are presented in Table 2, in relation to gestational age. There was no difference in cytokines levels between infants who received antibiotics for less and more than five days. IL-1Ra and IL-6 showed higher and more variable values in the first four hours after birth compared to the period thereafter, no trend over time was found during the rest of the first week. No trend over time was found in any of the other interleukines. Levels of IL-1b, IL-2r, IL-4, IL-5, IL-7, IFNa, GM-CSF, MIP-1b, Eotaxin and RANTES were not different at any time point between different gestational age groups. Levels of IL-2, IL-15, IL-17 and TNFα were lower in the infants born at 30-32 wks compared to the group > 36 wks at 4 and 24–72 hours. In the period 24–72 hours, levels of IL-1Ra, IL-6, IL-8, IL-12, IL-13, IFNγ, MIP-1a, IP-10 and MCP-1 were lower in the group 30-32 wks compared to ≥36 wks. Levels of IL-6, IL-10 and IP-10 were lower in the period 24–72 hours in the group 30–32 wks compared to the older infants. MIG was lower at birth in infants 30–32 weeks compared to the other groups.

**Table 2:** Serum concentrations of 25 cytokines and chemokines (pg/ml) between 3 study groups during first week of life

**Cytokinec/chemokines**			**GROUP**					
**After birth hours**			**≤ 32 wks (n = 11)**		**33 - 35 wks (n = 14)**		**≥ 36 wks (n = 9)**	
1.	IL1B:							
	-	0	0	(0–15)	1	(0–23)	7	(0–17)
	-	4	0	(0–4)	1	(0–30)	4	(0–20)
	-	24-72	0	(0–1)	1	(0,25-7,75)	4	(0,5-23)
	-	96-168			4	(0,75-17)	4	(0–14)
2.	IL1RA:							
	-	0	413	(158–931)	512	(179–22246)	803	(200–2376)
	-	4	2026	(63–13795)	596	(119–10464)	4183	(331–14702)
	-	24-72	242	(168–354)^@^&	508,5	(737,3-302,5)	545	(407–698)
	-	96-168			395,5	(309,8-556,8)	441	(179–613)
3.	IL2:							
	-	0	3,5	(3–7)	4	(1–15)	14	(3–18)
	-	4	3	(3–5)^@^	4	(1–17)	14	(4–17)
	-	24-72	3.5	(3–6.75)^@^	4	(3–10,5)	14	(5–15,5)
	-	96-168			9,5	(3–16,5)	14	(13–16)
4.	IL2R:							
	-	0	325	(236–427)	365	(202–604)	295	(145–507)
	-	4	300,5	(219–443)	349	(179–621)	295	(212–604)
	-	24-72	458	(332–617)	427	(268,5-556)	507	(387,5-631,5)
	-	96-168			692	(356,5-846)	610	(572–652)
5.	IL4:							
	-	0	2	(0–5)	5	(2–112)	2	(1–3)
	-	4	2	(0–5)	5	(2–128)	5	(1–10)
	-	24-72	2	(0–2)	4	(2–94)	3	(1–5,5)
	-	96-168			4,5	(2–22)	3	(1–6)
6.	IL5:							
	-	0	2	(0–2)	5	(0–34)	6	(0–8)
	-	4	2	(0–3)	5	(0–33)	6	(0–8)
	-	24-72	2,5	(2–4)	5	(2–7)	6	(2,3-8)
	-	96-168			8,5	(6–9,5)	6	(6–7)
7.	IL6:							
	-	0	15	(0–1440)	30,5	(1–418)	79	(64–232)
	-	4	12	(1–251)	26	(1–212)	94	(7–3135)
	-	24-72	4	(1,75-11)^@^	5,5	(2–16)^@^	17	(3–38)
	-	96-168			9,5	(5–29)	3	(2–8,8)
8.	IL7:							
	-	0	0	(0–0)	6	(0–40)	0,5	(0–1)
	-	4	0	(0–12)	3	(0–32)	0,5	(0–3)
	-	24-72	0	(0–0)	0	(0–8)	0	(0–6)
	-	96-168			3,5	(0–13,5)	0	(0–5,5)
9.	IL8:							
	-	0	57	(8–204)	98,5	(1–396)	40	(34–46)
	-	4	28,5	(10–157)	46	(1–189)	82,5	(13–189)
	-	24-72	19,5	(12,7-39,7)^@^&	44	(22,75-85,5)	57	(28–170)
	-	96-168			44,5	(24–78,25)	37,5	(13–64,3)
10.	IL10:							
	-	0	3	(3–29)	14,5	(0–74)	22	(22–25)
	-	4	3,5	(1–28)	9	(1–49)	23	(2–48)
	-	24-72	3	(2–3)^@^	3	(2–11)^@^	21	(3,3-22)
	-	96-168			14	(3–21)	21	(3–21,8)
11.	IL12:							
	-	0	418,5	(179–693)	473	(215–815)	278	(202–1837)
	-	4	461,5	(144–628)	345	(171–960)	1151	(258–1883)
	-	24-72	263	(225–339)^@^&	384	(199,8-517,8)	402	(295,5-499,3)
	-	96-168			358	(243,8-445,8)	431	(219–602)
12.	IL13:							
	-	0	0	(0–7)	7	(0–26)	12	(0–70)
	-	4	0	(0–7)	1	(0–22)	14,5	(0–81)
	-	24-72	0	(0–7)^@^	1,5	(0–8,5)	12	(0–25)
	-	96-168			7	(0–17)	17	(12–26)
13.	IL15:							
	-	0	0	(0–0)	13	(0–64)	37	(0–49)
	-	4	0	(0–0)^@^&	10	(0–49)	39	(0–53)
	-	24-72	0	(0–0)^@^&	13	(0–42)	41	(0–53)
	-	96-168			39,5	(0–52)	37	(28–47)
14.	IL17:							
	-	0	0	(0–0)	36	(0–130)	23	(0–33)
	-	4	0	(0–6)^@^&	26	(0–146)	22	(6–38)
	-	24-72	0	(0–1)^@^&	27,5	(0,8-44,8)	18	(8,5-31,5)
	-	96-168			29	(4,5-41,8)	19	(0–28)
15.	TNFA:							
	-	0	0	(0–0)	1	(0–6)	3	(0–6)
	-	4	0	(0–0)^@^&	1	(0–7)	3	(0–5)
	-	24-72	0	(0–0)^@^&	1	(0–3,8)	3	(0–4,5)
	-	96-168			4	(0–6,3)	4	(0–4)
16.	IFNA:							
	-	0	36	(20–49)	40	(18–88)	30	(8–58)
	-	4	20	(0–49)	36	(18–98)	49	(20–79)
	-	24-72	36	(22,5-49)	49	(26–64,5)	36	(30–44,5)
	-	96-168			44,5	(36–75)	40	(30–49)
17.	IFNG:							
	-	0	3	(3–3)	8	(2–449)	7	(3–8)
	-	4	3	(2–4)	8	(3–603)	7	(3–9)
	-	24-72	3	(3–3,75)^@^&	7,5	(3–43)	7	(4–8,5)
	-	96-168			8	(4–15,5)	7	(7–8)
18.	GM_CSF:							
	-	0	0	(0–0)	1	(0–48)	0	(0–1)
	-	4	0	(0–0)	1	(0–27)	0	(0–34)
	-	24-72	0	(0–0)	0	(0–2)	0	(0–10,5)
	-	96-168			1	(0–22)	0	(0–7)
19.	MIP1A:							
	-	0	24	(22–32)	28	(23–150)	28	(19–34)
	-	4	23	(22–34)	26	(18–150)	30	(23–36)
	-	24-72	23,5	(23–25)^@^&	29,5	(25–36,8)	28	(26–37)
	-	96-168			30	(25–38,8)	26	(24–32)
20.	MIP1B:							
	-	0	82	(23–191)	74	(34–345)	40	(22–237)
	-	4	74	(51–133)	66	(41–550)	83	(57–250)
	-	24-72	41,5	(21–110,75)	80	(54–145,5)	66	(47,3-120,3)
	-	96-168			86,5	(45,5-149,3)	60	(42–90)
21.	IP10:							
	-	0	19	(8–144)	32,5	(8–85)	115	(16–124)
	-	4	29,5	(3–162)	29	(6–119)	72,5	(19–119)
	-	24-72	24,5	(16–54)^@^	37	(18,8-56,3)	82,5	(47–136,8)
	-	96-168			61	(39–140,3)	55,5	(34–133,75)
22.	MIG:							
	-	0	32	(23–71)^@^	41	(8–118)	0	(0–8)
	-	4	23	(0–41)	23	(0–145)	8	(0–47)
	-	24-72	23	(23–41)	35,5	(23–57)	41	(2–47)
	-	96-168			61	(39–140,3)	23	(8–47)
23.	EOTAXIN:							
	-	0	24	(14–37)	35	(10–221)	9	(4–40)
	-	4	30,5	(11–80)	33	(10–86)	39	(12–220)
	-	24-72	30	(16,5-65)	51	(36,5-81,5)	32,5	(26,3-61,5)
	-	96-168			54	(28,5-67,5)	41	(25–55)
24.	RANTES:							
	-	0	1200	(1200–4967)	1200	(1000–18288)	1000	(1000–1200)
	-	4	1200	(1200–9900)	1200	(1000–18248)	1000	(1000–1443)
	-	24-72	1200	(1200–2821)	1200	(1200–10608,8)	1000	(1000–1200)
	-	96-168			1100	(1000–1718,8)	1000	(1000–1132)
25.	MCP1:							
	-	0	439,5	(234–3238)	399	(120–2252)	163	(69–2786)
	-	4	355,5	(0–3841)	433	(84–1685)	1119	(210–11314)
	-	24-72	275,5	(141–516)^@^	463	(319,8-836)	934	(401–3650)
	-	96-168			378	(270,8-650,3)	406	(189–879)

Data shown as median (min-max).

Used Mann Whitney U test.

^@^: p < 0.01; comparison between group ≤32 wks / 33–35 wks and group term.

&: p < 0.01; comparison betweeen group ≤32 wks and group 33–35 wks.

## Discussion

In this paper we show that levels of 25 cytokines/chemokines in non-infected newborn infants are constant between day 2 and 7 in both preterm and term infants. Levels of IL-1Ra, IL-2, IL-6, IL-8, IL-10, IL-12, IL-13, IL-15, IL-17, TNFα, IFNγ, MIP-1a, IP-10 and MCP-1 are lower in infants born at 30-32wks compared to infants born at or ≥36 wks at day two and three days of life. IL-6, IL-10 and IP-10 were also lower in the group born between 33 and 35 weeks compared the group ≥36 wks.

Matoba et al. [14] recently described levels of 27 immune biomarkers in cord blood. Concentrations of 12 biomarkers (IL-2, IL-4, IL-5, IL-8, IL-10, MCP-1, MIP-1a, MIP-1b, sIL-6ra, sTNF-RI, TNFα and TREM-1) were higher in preterm compared to term infants, while IL-1b and IL-18 were lower. These authors assumed that the higher level of a number of cytokines were related to preterm birth. It is not sure however if preterm birth itself caused an increased level in cytokines or that a common factor like an infection caused both an increase in cytokines level and preterm birth. The incidence of chorioamnionitis for instance was higher in group preterm compared to term infants. Levels of the pro-inflammatory cytokines MIP-1a and MIP-1b showed the highest correlation between gestational age and cytokines levels. Preliminary data from studies conducted by us as well as other studies indicate that MIP1a and MIP1b are specific markers for a bacterial infection. The study of Matoba did not provide data on blood cultures in the infants, also no data on cytokine levels during the first week of life. A recent study by McElrath et al. [17] showed that cytokines measured on the first day of life were higher in preterm infants born after complications that are associated with infections compared to preterm infants born after complications like pre-ecclampsia. Blanco-Quiros [18] observed higher values of IL-10, but not of IL-12 in preterm compared to term infants. The higher levels of IL-10 were mainly found in infants less than 30 weeks, not included in our study. Moreover, they analysed cord blood while we took our first sample from the infant itself. Dembinski et al. [19] did not find an influence of gestational age on IL-10 levels in cord blood. In that study only IL-6 and GM-CSF were higher in cord blood in term vs. preterm infants.

Our study has a number of limitations. The number of infants included into the study is relatively small. It is difficult however to take sequential, daily, blood samples in healthy preterm and term infants. Therefore we included infants who after birth were on N-CPAP that needed to take blood samples. Secondly, we did not include infants of less than 30 weeks. At the start of the study we realized that the number of these infants admitted to our unit is too small to be able to include a sufficient number in this study. Finally, a limitation of our study is that we included infants who did receive CPAP and therefore might be not considered as completely healthy infants. There were no other signs that made them suspected of having an infection. All infants included in this study received antibiotics, as it is practice in our unit to prescribe antibiotics to all infants showing respiratory problems at birth. Still, all infants were stable during the whole first week of life, not showing signs indicating an infection. In all infants a blood culture was taken on admission and found to be negative. We are convinced therefore that all included infants did not have an infection. Our definition of the non infected group is consistent with the definition used by Ng et al. [9].

In conclusion, levels of 25 interleukines are stable in the first week of life in non-infected infants. Infants born after 30-32 wks showed lower levels of fourteen cytokines compared to infants born after more then 36 wks. This indicates a lower stimulation or activation of Th-1 cells, monocytes and dendritic cells in these infants.

## Abbreviations

NICU: Neonatal Intensive care unit; IL: Interleukin; AS: Apgar score; TNFα: Tumor necrosis factor alpha; IFNγ: Interferon gamma; CRP: C-reactive proteine; TH: T-helper; MCP1: Monocyte chemoattaractant proteine-1; MIP1a/b: Macrophage inflammatory proteine-1a/b

## Competing interests

The authors declare that they have no competing interests.

## Authors’ contributions

SL designed the study, performed the study and wrote the paper. CH designed the study, supervised the cytokine analysis and reviewed the paper. JZ performed all cytokine analysis. PS Supervised the study design, the analysis of the data and wrote the paper. All authors read and approved the final manuscript.
